# Personal

**Published:** 1896-01

**Authors:** 


### Personal.

---

Dr. Herman G. Matzinger has severed his relation with the Buffalo State Hospital and has opened an office at 519 Franklin Street, Buffalo. He will give special attention to mental and nervous diseases.

---

Dr. W. G. Grove, of Buffalo, has removed his office and residence from 197 West Utica street to 89 Niagara street. Hours, 8–10 a. m., 1–3 and 7 p. m. Telephone, Seneca, 1022.

---

Dr. John S. Billings, Deputy Surgeon-General U. S. Army (retired), has been appointed professor of hygiene in the University of Pennsylvania.

---

Dr. and Mrs. Leon F. Harvey, of Buffalo, have gone to Southern California for the purpose of enjoying its genial climate during the winter.

---

Dr. George J. Engelmann has removed from St. Louis to Boston. His office is at 336 Beacon Street. Hours, 2–3.30.

